# The relationship between T-lymphocyte infiltration, stage, tumour grade and survival in patients undergoing curative surgery for renal cell cancer

**DOI:** 10.1038/sj.bjc.6601400

**Published:** 2003-11-11

**Authors:** E J Bromwich, P A McArdle, K Canna, D C McMillan, A-M McNicol, M Brown, M Aitchison

**Affiliations:** 1 Department of Urology, Gartnavel General Hospital, Glasgow G12 0YN, UK; 2 University Departments of Surgery, Royal Infirmary, Glasgow G31 2ER, UK; 3 University Department of Pathology, Royal Infirmary, Glasgow G31 2ER, UK

## Abstract

The present study examined the relationship between tumour stage, grade, T-lymphocyte subset infiltration and survival in patients who had undergone potentially curative surgery for renal clear-cell cancer (*n*=73). Intratumoural CD4+ T-lymphocyte infiltrate was associated with poor cancer-specific survival, independent of grade, in this cohort.

Local and systemic inflammatory responses are regulated through the production of proteins, such as cytokines, by immunologically active cells. In cancer, this mechanism is disturbed by the presence of the tumour and this dysregulation may contribute to the poorer outcome in the cancer patient (Balkwill and Mantovani, 2001).

Recent work has suggested that the presence of specific T-lymphocyte subsets has prognostic value in a number of solid tumours. Naito *et al* (1998) demonstrated that increased numbers of CD8+ T lymphocytes in the tumour were associated with better survival in patients with colorectal cancer.

There has been little work carried out in renal cancer and the results appear to be different from other solid tumours. Kolbeck *et al* (1992) in a small study reported that increased tumour T-cell infiltration was associated with increased tumour recurrence. More recently, in a larger series, it has been reported that increased numbers of CD8+ T lymphocytes in the tumour are associated with poor survival in patients with renal cancer (Nakano *et al*, 2001).

The aim of the present study was to examine the relationship between tumour stage, grade, T-lymphocyte subset infiltration and survival in patients who had undergone potentially curative surgery for renal clear-cell cancer.

## PATIENTS AND METHODS

### Patients

Patients with histologically proven renal clear-cell cancer that, on the basis of preoperative CT-scan of the abdomen and chest and pathological assessment of the resected tumour, were considered to have undergone potentially curative surgery between July 1997 and December 2000 in the North Glasgow NHS Trust were included in the study. Pathological staging was based on TNM and classified as ⩽II or >II (Guinan *et al*, 1997).

The study was approved by the local ethical committee.

### Immunohistochemistry

Blocks from the primary tumour were fixed in 10% buffered formalin and embedded in paraffin wax. One representative block of tumour was selected for each patient. Sections (4 *μ*m) were cut and mounted on slides coated with aminopropyltriethoxysilane.

Sections were then immunostained using the peroxidase-based Envision (Dako, Cambridgeshire, UK) technique. The primary antibody for CD4 was mouse monoclonal (Vector, Peterborough, UK) and that for CD8 was mouse monoclonal (Dako, Cambridgeshire, UK). Sections were dewaxed and rehydrated. Endogenous peroxidase was blocked by incubation in 0.3% hydrogen peroxide for 10 min. Antigen retrieval for CD8 was performed by microwaving in 1 mM EDTA buffer, pH 8, for 5 min at full pressure in aplastic pressure cooker in a microwave oven. Antigen retrieval for CD4 was achieved by immersing the sections in high pH buffer (9.9, Dako) in a Coplin jar, maintained at 99°C for 75 min in a water bath.

The sections were then incubated with the primary antibodies at dilutions of 1 : 50 (CD4) and 1 : 100 (CD8) for 30 min at room temperature. Sites of binding were detected using the Envision kit with 3′3′diaminobenzidine as chromogen according to the manufacturer's instructions. Sections were counterstained with haematoxylin, dehydrated, cleared and mounted with Pertex.

### Morphometry

Quantitative analysis of the lymphoid infiltrate was performed using point counting (Anderson and Dunnill, 1965) with a random sampling technique. With this method, the volume occupied by any given component (volume density) is expressed as a percentage of the total volume of the tissue. In the present study, the volumes of CD4+ and CD8+ immunopositive cells were calculated as percentage of the total tumour volume. A 100-point ocular grid was used at × 400 magnification and 30 fields were counted per case for each antibody. Only fields within the tumour (including cancer cell nests and surrounding tissue stroma) were counted. Any normal tissue on the slide was excluded from the analysis.

This final method was designed on the basis of a pilot study, which demonstrated that the volume density of CD4+ and CD8+ of two observers reached a plateau after 25–30 fields. This pilot study also demonstrated that CD4+ and CD8+ counts were equivalent to the CD3+ counts (unpublished data). The observers (KC and PAM) were blinded to the clinical outcome of the patient.

### Statistics

The data are presented as median and range. Comparisons between groups of patients were carried out using contingency table analysis (*X*^2^), the Mann–Whitney *U*-test or Kaplan–Meier analysis as appropriate.

Survival analysis was performed using the Cox proportional hazard model with patients' age, sex, TNM stage, CD4+ and CD8+ count as prognostic variables. Deaths up to September 2002 have been included in the analysis. Multivariate survival analysis was performed using a stepwise backward procedure to derive a final model of the variables that had a significant independent relationship with survival. To remove a variable from the model, the corresponding *P*-value had to be greater than 0.10. Analysis was performed using SPSS software (SPSS Inc., Chicago, IL, USA).

## RESULTS

Baseline characteristics of the patients (*n*=73) who underwent curative surgery for renal cell cancer are shown in Table 1

**Table 1:** Baseline characteristics, according to stage, of patients who underwent curative surgery for renal cancer

	**TNM stage ⩽II**	**TNM stage >II**	
	**(*n*=40)**	**(*n*=33)**	***P*-value**
Age group			
⩽60	22	14	
>60	18	19	0.285
Sex			
Male	26	23	
Female	14	10	0.671
			
Grade Furhman			
I	10	2	
II	20	10	
III	7	13	
IV	3	8	0.007
Tumour volume (cm^3^)	69 (1–3040)	224 (21–729)	0.003
% tumour volume			
CD4+^a^	0.40 (0.07–1.63)	0.70 (0.13–2.10)	0.046
CD8+^a^	1.08 (0.03–6.13)	0.97 (0.20–9.40)	0.833
CD4+ plus CD8+^a^	1.40 (0.10–6.73)	1.67 (0.40–11.4)	0.303
			
Alive	33	18	
Dead (cancer specific/noncancer)	4/3	13/2	0.012
Median survival (months)	29.1 (15.4–35.6)	19.2 (2.1–28.1)	0.002

a Median (range).

. The majority of patients was male, had stage I/ II disease and had grade II/ III tumours. Compared with patients of stage <II disease, tumours were of higher grade (*P*<0.01), greater volume (*P*<0.01) and increased CD4+ T-lymphocytic infiltration (*P*<0.05) in patients with stage >II disease. This was also associated with poorer cancer-specific survival (*P*<0.01).

No patients received additional therapy in the immediate postoperative period. A total of 17 patients developed recurrence and all received alpha-interferon based immunotherapy.

On univariate analysis, TNM stage (*P*<0.01), grade (*P*<0.001) and CD4+ count (*P*<0.001) were associated with cancer-specific survival (Table 2

**Table 2:** Univariate analyses of the relationship between variables and cancer-specific survival in renal cancer patients following curative resection

**Variable**	**Hazard ratio (95% CI)**	***P*-value**
Age (⩽60/>60 years)		0.939
Sex (male/female)		0.177
TNM stage (⩽II/>II)	5.05 (1.64–15.52)	0.005
Grade (Fuhrman I/II/III/IV)	2.60 (1.48–4.56)	<0.001
		
Tumour volume (cm^3^)		0.885
% tumour volume		
CD4+	4.96 (2.13–11.55)	<0.001
CD8+		0.405

). On multivariate analysis, grade (HR 2.47, 95% CI 1.37–4.46, *P*<0.01) and percentage CD4+ (HR 4.44, 95% CI 1.87–10.57, *P*<0.001) were independently associated with cancer-specific survival.

## DISCUSSION

The results of the present study show that an increase in CD4+ but not CD8+ T-lymphocyte infiltrate, as assessed by immunohistochemistry, was associated with poor cancer-specific survival, independent of grade, in patients undergoing potentially curative surgery for renal clear-cell cancer. Few studies have examined the relationship between tumour T-lymphocyte infiltrate and outcome in patients with renal cancer. Kolbeck *et al* (1992) reported that, in a small study of 24 cases, increased tumour T-cell infiltration was associated with increased tumour recurrence. Nakano *et al* (2001) reported that a high tumour CD8+ T-lymphocyte infiltrate was associated with poorer survival independent of grade. In contrast, in the present study, there was no relationship between tumour CD8+ T-lymphocyte infiltrate and cancer-specific survival. This discrepancy may be related to the larger number of cases in their study. Alternatively, it may be due to methodological differences in the way in which the T-cell infiltrate was calculated. The sampling in the present study was much greater and was designed to circumvent the problem of variation in distribution of lymphocytes within an individual tumour. Nakano *et al* (2001) did not specifically quantify intratumoural CD4+ lymphocytes and therefore direct comparison with the present study may be inappropriate. However, they performed a semiquantitative analysis of CD4+ and CD8+ lymphocytes at the periphery of the tumours and found that higher levels of infiltration of both were associated with reduced survival. We have not looked specifically in this area.

The findings of the present and previous studies would suggest that a conspicuous tumour T-lymphocyte infiltrate, in particular CD4+, was associated with poor cancer survival in patients with renal cell cancer. However, it is not clear whether the infiltration of CD4+ T-lymphocytes within the tumour is a sign of an active immune response or whether it is a more passive consequence of cytokine excretion from the tumour that attracts T lymphocytes. Given that renal cancer is recognised to be immunogenic in nature and that it responds to immunomodulatory therapy, it may be speculated that the immune response in renal cancer is an active, perhaps antigen driven, process (Michael and Pandha, 2003).

In summary, we have shown that increased intratumoural CD4+ T-lymphocyte infiltrate was associated with poor outcome, independent of grade, in patients with renal clear-cell cancer.
